# USP22 regulates APL differentiation via PML-RARα stabilization and IFN repression

**DOI:** 10.1038/s41420-024-01894-8

**Published:** 2024-03-11

**Authors:** Lisa Kowald, Jens Roedig, Rebekka Karlowitz, Kristina Wagner, Sonja Smith, Thomas Juretschke, Petra Beli, Stefan Müller, Sjoerd J. L. van Wijk

**Affiliations:** 1 Institute for Experimental Pediatric Hematology and Oncology, Medical Faculty, Goethe-University Frankfurt, Komturstrasse 3a, 60528 Frankfurt am Main, Germany; 2 Institute of Biochemistry II (IBCII), Medical Faculty, Goethe University Frankfurt, Theodor-Stern-Kai 7, 60590 Frankfurt am Main, Germany; 3 Institute of Molecular Biology (IMB), Ackermannweg 4, 55128 Mainz, Germany; 4 German Cancer Consortium (DKTK), partner site Frankfurt/Mainz, Frankfurt am Main, Germany; 5 German Cancer Research Center (DKFZ), Heidelberg, Germany; 6 University Cancer Centre Frankfurt (UCT), University Hospital Frankfurt, Goethe-University Frankfurt, Frankfurt, Germany

**Keywords:** Cell biology, Cancer

## Abstract

Ubiquitin-specific peptidase 22 (USP22) is a deubiquitinating enzyme (DUB) that underlies tumorigenicity, proliferation, cell death and differentiation through deubiquitination of histone and non-histone targets. Ubiquitination determines stability, localization and functions of cell fate proteins and controls cell-protective signaling pathways to surveil cell cycle progression. In a variety of carcinomas, lymphomas and leukemias, ubiquitination regulates the tumor-suppressive functions of the promyelocytic leukemia protein (PML), but PML-specific DUBs, DUB-controlled PML ubiquitin sites and the functional consequences of PML (de)ubiquitination remain unclear. Here, we identify USP22 as regulator of PML and the oncogenic acute promyelocytic leukemia (APL) fusion PML-RARα protein stability and identify a destabilizing role of PML residue K394. Additionally, loss of USP22 upregulates interferon (IFN) and IFN-stimulated gene (ISG) expression in APL and induces PML-RARα stabilization and a potentiation of the cell-autonomous sensitivity towards all-*trans* retinoic acid (ATRA)-mediated differentiation. Our findings imply USP22-dependent surveillance of PML-RARα stability and IFN signaling as important regulator of APL pathogenesis, with implications for viral mimicry, differentiation and cell fate regulation in other leukemia subtypes.

## Introduction

By selectively removing ubiquitin molecules, the deubiquitinating enzyme (DUB) USP22 controls gene expression via histone modification and stabilization of transcriptional activators or repressors [1–4]. Apart from deubiquitinating histones H2A and H2B, USP22 also modulates transcription through stabilization of the p53-antagonizing deacetylase sirtuin-1 (SIRT1) to suppress p53-controlled transcription and to abrogate pre-mature senescence in pluripotent progenitor cells during embryonic development [5]. High USP22 expression levels potently suppress the transcriptional activity of p53 that contributes to cancer cell proliferation and inhibition of apoptosis or senescence [6]. USP22-mediated suppression of p53 is counteracted by the promyelocytic leukemia (PML) protein that enhances senescence and apoptosis signaling by acetylating p53 [7], and interferes with SIRT1 and the E3 ubiquitin ligase MDM2 [8]. USP22 is highly expressed in several cancer types and USP22 abundance is linked to disease severity by promoting oncogenic gene expression, aberrant cell cycle regulation and cancer cell proliferation [9–11]. On the other hand, PML expression is reduced in many tumor types [12], favoring pro-tumorigenic effects of USP22 on cell cycle progression, but it remains unclear how USP22 regulates the tumor-associated functions of PML.

PML controls many cellular processes, including cell cycle regulation, transcriptional and translational modification and cellular stress response signaling via protein-protein interactions, protein sequestration via compartmentalization or by DNA and histone engagement [13]. These diverse functions originate from an array of PML isoforms (I–VII, and additional splice variants), that are located in different cellular compartments with dedicated domain compositions [14]. For example, PML isoform IV controls senescence and apoptosis via p53 [15] and cytoplasmatic PML isoforms regulate extrinsic death receptor-mediated apoptosis signaling [16], while the most abundant isoform PML I is associated with angiogenesis in neuroblastoma [17] and PML isoform V serves as structural scaffold of PML nuclear bodies (NBs) [18]. In addition, a genetic fusion of the N-terminal part of PML on chromosome 15 with the C-terminal ligand- and DNA-binding domain of the retinoic acid receptor-α (RARα) on chromosome 17 gives rise to PML-RARα chimeric proteins with breakpoints at either PML lysine (K) 394 or alanine (A) 552 [19].

PML-RARα is an oncogenic driver in acute promyelocytic leukemia (APL), where it acts as a transcriptional repressor of RARα-targeted granulocyte differentiation genes, leading to a persistence of leukemic progenitor blasts (reviewed in detail by [20]). Standard-of-care treatment of APL includes arsenic trioxide (ATO) for proteasomal degradation of PML-RARα as well as the endogenous RARα ligand all-*trans* retinoic acid (ATRA), that provokes a strong remission of the disease via two major molecular mechanisms [20]. First, high therapeutic doses (0.1–1 µM) of ATRA alter the repressive functions of the chimeric PML-RARα protein into transcriptional activation of genes involved in terminal myelocyte differentiation [21]. Second, ATRA facilitates PML-RARα proteasomal degradation via caspase-mediated cleavage and cyclic adenosine monophosphate (cAMP)-mediated degradation through ligand-binding domain phosphorylation [22–24]. Although both mechanisms are considered individual modes of action, both are required for APL remission, since terminally differentiated but PML-RARα-persistent myelocytes are prone to reinitiate APL [25].

The activity of PML and PML-RARα is tightly controlled by post-translational modifications. PML residues K65, K160 and K490 are modified with small ubiquitin-like modifier (SUMO) [26] that allows PML multimerization into NBs [27]. Several other PML residues are subjected to SUMOylation, acetylation or phosphorylation, that regulate PML cellular localization, oligomerization and association with effector proteins [28–30]. PML is heavily modified with ubiquitin and more than ten ubiquitin acceptor lysine residues have been identified on PML and several E3 ligases, like ubiquitin-protein ligase E3A (UBE3A or E6AP), seven in absentia homolog 1 and 2 (SIAH-1/2) or Kelch-like protein 20 (KLHL20) [31–33] regulate basal PML turnover. Degradation of PML induces pro-tumorigenic phenotypes, characterized by cell survival and cell cycle dysregulation [34], but it remains largely unclear how PML functions are regulated by DUBs.

Here, we identify USP22 as novel regulator of basal and tumor-associated PML and PML-RARα function, interferon (IFN) regulation and APL differentiation. We demonstrate that USP22 controls basal stability of tumor-suppressive PML and identify a destabilizing role of PML K394. In addition, USP22 also determines protein stability of the oncogenic PML-RARα fusion protein and regulates ATRA-mediated APL differentiation. Apart from that, our work shows that USP22 negatively regulates IFN and IFN-stimulated gene (ISG) signaling in APL, which is a critical determinant of ATRA-induced APL differentiation. These findings provide valuable and novel insights into USP22-mediated tumorigenicity and elucidate potential implications against relapsed and therapy-resistant APL.

## Results

### USP22 regulates PML stability

**Fig. 1:**
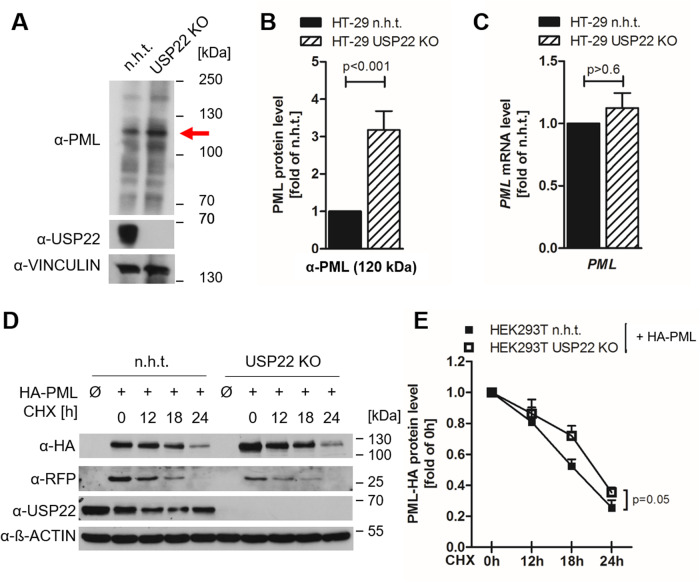
USP22 controls PML expression. **A** Western blot analysis of basal PML and USP22 expression in control (non-human target; n.h.t) and USP22 knockout (KO) HT-29 cells. The major (120 kDa) PML isoform is indicated with a red arrow. Vinculin served as loading control. Representative blots of at least two different independent experiments are shown. **B** Densitometric quantification of gray level intensities of the major (120 kDa) PML isoform (indicated with red arrow) detected by Western blot analysis of PML in n.h.t and USP22 KO HT-29 cells from (**A**), normalized against loading control intensities. Mean and SEM of three independent experiments are shown. **C** Basal mRNA expression levels of all PML isoforms in n.h.t and USP22 KO HT-29 cells using qRT-PCR. Gene expression was normalized against 28S mRNA and is presented as x-fold mRNA expression compared to n.h.t. Mean and SEM of three independent experiments are shown. **D** Western blot analysis of n.h.t and USP22 KO HEK293T cells transiently transfected with plasmids expressing PML-HA isoform IV and mCherry, prior to treatment with 20 μg/ml cycloheximide (CHX) for the indicated timepoints. β-Actin served as loading control. Representative blots of at least three different independent experiments are shown. **E** Densitometric quantification of gray level intensities of PML-HA isoform IV detected by Western blot analysis of HA in n.h.t and USP22 KO HEK293T cells in the presence of CHX for the indicated timepoints, normalized against loading control intensities. Mean and SEM of three independent biological replicates are shown.

Colorectal carcinomas represent tumor entities in which USP22 expression is well correlated with malignant tumor progression [35, 36]. PML serves as tumor suppressor and loss, or reduced expression, of PML allows additional tumorigenic mutations to drive the formation of solid tumors, including colon carcinomas [12]. Here, the functional consequences of USP22 on PML expression levels were investigated by CRISPR/Cas9-mediated constitutive knock-out (KO) of USP22 in the human HT-29 colon carcinoma cell line, generated as described previously [37]. Comparative immunoblot analysis of denatured whole cell lysates revealed the expression of a variety of PML isoforms with different molecular weights (Fig. 1A), reflecting the well-described alternative splicing events of the *PML* gene locus in up to 14 different transcripts [14]. Among these, the expression of putative PML isoform I of approximately 120 kDa was increased up to 3-fold in USP22 KO cells compared to control cells (Fig. 1A, B). Of note, the increased PML protein abundance is most likely not caused by transcriptional regulation, since the basal mRNA expression levels of all PML isoforms were not significantly altered upon loss of USP22 expression (Fig. 1C).

To address the potential role of USP22 in regulating PML stability, equal amounts of HA-tagged PML isoform IV (HA-PML) were co-expressed with mCherry transfection controls in control and USP22 KO HEK293T human embryonic kidney cells. Intriguingly, despite equal amounts of transfected plasmid DNA, USP22 KO HEK293T cells demonstrate a slight tendency of higher HA-PML expression levels compared to control cells (Fig. 1D). In addition, blockade of protein translation with cycloheximide (CHX) revealed a slightly faster degradation of PML isoform IV in USP22 KO cells compared to control cells **(**Fig. 1D, E). While the normalized protein HA-PML half-life was *t*_1/2_ = 18 h in control cells, HA-PML turnover was prolonged to approximately *t*_1/2_ = 21 h upon loss of USP22 (Fig. 1E). Of note, the half-life of co-expressed mCherry was not differentially affected in control and USP22 KO cells, excluding global USP22-mediated effects on protein translation, degradation and stabilization (Supplementary Fig. 1). These findings reveal that USP22 might affect PML turnover.

### Residue K394 affect PML stabilization

The post-translational modification of proteins with ubiquitin is an important determinant for regulating protein stability, localization and function [38]. To further investigate the link between USP22 and PML ubiquitination, a recently published mass spectrometry dataset was analyzed for USP22-dependent changes in ubiquitinated peptides in control and USP22 KO HT-29 cells (see [37] and Supplementary Fig. 2A). In agreement with previous findings that report PML ubiquitination under homeostatic conditions [39], an approximately 2-fold USP22-dependent increase in ubiquitinated PML K394 peptide could be detected, compared to control HT-29 cells (Supplementary Fig. 2B–D).

**Fig. 2:**
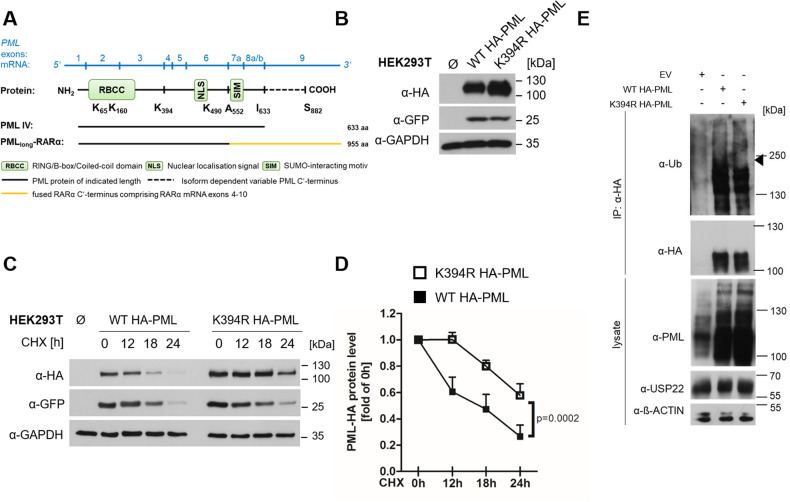
PML lysine 394 is required for maintaining USP22-dependent PML stability. **A** Schematic overview of PML mRNA (blue) with approximate exon spans indicated, as well as the PML (black) and PML-RARα protein (yellow) with relevant amino acids indicated (K, lysine; A, alanine; I, isoleucine; S, serine). RBCC/Trim motif, RING-B-box1-B-box2-Coiled-Coil domain; NLS, nuclear localization signal; SIM, SUMO-interacting motif. **B** Western blot analysis of HEK293T cells transiently transfected with plasmids expressing wild-type (WT) and K394R HA-tagged PML isoform IV. Co-transfection with GFP plasmid served as transfection control. GAPDH served as loading control. Representative blots of at least two different independent experiments are shown. **C** Western blot analysis of HEK293T cells transiently transfected with plasmids expressing WT and K394R HA-PML isoform IV, prior to treatment with 20 μg/ml CHX for the indicated timepoints. Co-transfection with GFP plasmid served as transfection control. GAPDH served as loading control. Representative blots of at least two different independent experiments are shown. **D** Densitometric quantification of gray level intensities of WT and K394R HA-PML isoform IV detected by Western blot analysis of HA in HEK293T cells in the presence of CHX for the indicated timepoints, normalized against loading control intensities. Mean and SEM of three independent biological replicates are shown. **E** Western blot analysis of anti-ubiquitin (Ub), -HA and -PML on anti-HA-immunoprecipitated fractions of denatured lysates of HEK293T cells transiently transfected with empty vector (EV) or plasmids expressing WT and K394R HA-PML isoform IV. ß-Actin served as loading control. Representative blots of at least two different independent experiments are shown.

PML K394 is located towards the end of PML exon 3 and is conserved in all known human PML isoforms (Fig. 2A). To analyze the functional relevance of K394 for controlling PML stability, K394 was mutated to arginine (R). Transient expression of HA-tagged K394R PML isoform IV in HEK293T cells resulted in an increased protein expression compared to HA-tagged wild-type (WT) PML isoform IV (Fig. 2B). Of note, co-expressed GFP transfection controls were equally expressed, making global effects on plasmid uptake, expression or translation unlikely (Fig. 2B).

Mutation of PML isoform IV K394 prolonged the half-life of PML isoform IV by approximately 9 h to *t*_1/2_ = 25.5 h, compared to a half-life of *t*_1/2_ = 16.5 h observed for WT HA-PML isoform IV (Fig. 2C, D). In addition, denaturing immunoprecipitations of K394R HA PML isoform IV revealed less ubiquitination compared to WT HA- PML isoform IV (Fig. 2E).

### Residue K394 affects PML-RARα stability

**Fig. 3:**
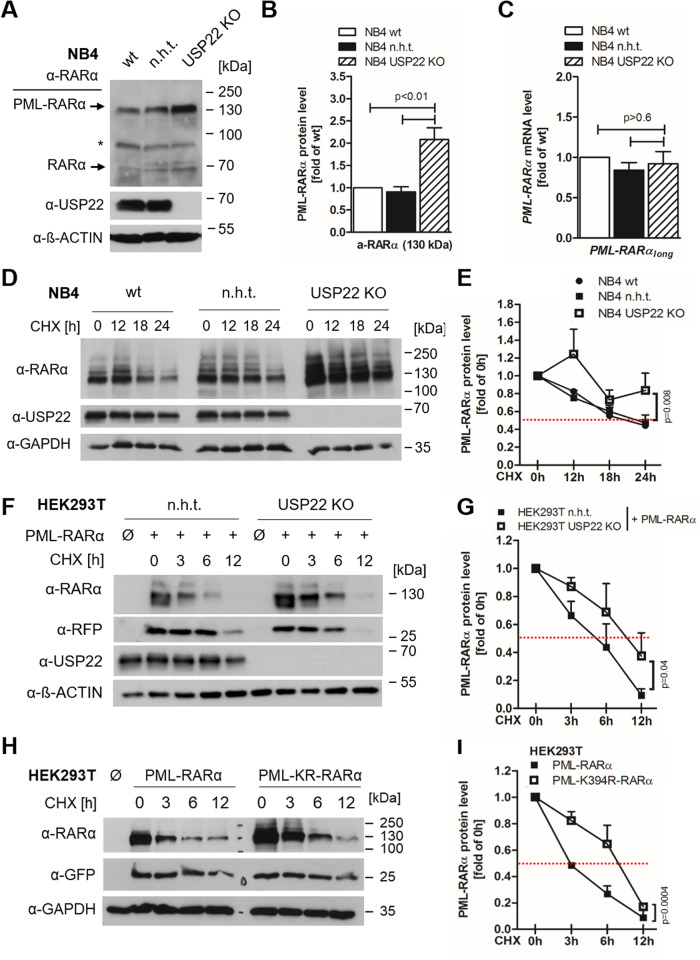
USP22 regulates the stability of PML-RARα. **A** Western blot analysis of basal PML-RARα and USP22 expression in wild-type (wt), control (non-human target; n.h.t) and USP22 knockout (KO) NB4 acute promyelocytic leukemia (APL) cells. β-Actin served as loading control. Representative blots of at least two different independent experiments are shown. **B** Densitometric quantification of gray level intensities of the major (130 kDa) PML-RARα isoform detected by Western blot analysis in wt, n.h.t and USP22 KO NB4 APL cells, normalized against loading control intensities. Mean and SEM of three independent experiments are shown. **C** Basal mRNA expression levels of the PML-RARα long isoform in wt, n.h.t. and USP22 KO NB4 APL cells using qRT-PCR. Gene expression was normalized against 28S mRNA and is presented as x-fold mRNA expression compared to n.h.t. Mean and SEM of three independent experiments are shown. **D** Western blot analysis of wt, n.h.t and USP22 KO NB4 APL cells treated with 20 μg/ml CHX for the indicated timepoints. GAPDH served as loading control. Representative blots of at least two different independent experiments are shown. Red dashed line indicates protein stability at 50% compared to untreated controls. **E** Densitometric quantification of gray level intensities of the major (130 kDa) PML-RARα isoform detected by Western blot analysis in wt, n.h.t and USP22 KO NB4 APL cells treated with 20 μg/ml CHX for the indicated timepoints, normalized against loading control intensities. Mean and SEM of three independent experiments are shown. **F** Western blot analysis of n.h.t and USP22 KO HEK293T cells, transiently transfected with plasmids encoding the long isoform of PML-RARα and treated with 20 μg/ml CHX for the indicated timepoints. Co-transfection with GFP plasmid served as transfection control. GAPDH served as loading control. Representative blots of at least two different independent experiments are shown. Red dashed line indicates protein stability at 50% compared to untreated controls. **G** Densitometric quantification of gray level intensities of the long isoform of PML-RARα transiently transfected in n.h.t and USP22 KO HEK293T cells treated with 20 μg/ml CHX for the indicated timepoints, normalized against loading control intensities. Mean and SEM of three independent experiments are shown. **H** Western blot analysis of HEK293T cells, transiently transfected with plasmids encoding the long isoform of wt (PML-RARα) and K394R (KR) PML-RARα (PML-K394R-RARα) and treated with 20 μg/ml CHX for the indicated timepoints. Co-transfection with GFP plasmid served as transfection control. GAPDH served as loading control. Representative blots of at least two different independent experiments are shown. Red dashed line indicates protein stability at 50% compared to untreated controls. **I** Densitometric quantification of gray level intensities of the long isoform of wt (PML-RARα) and K394R (KR) PML-RARα (PML-K394R-RARα) transiently transfected in HEK293T cells treated with 20 μg/ml CHX for the indicated timepoints, normalized against loading control intensities. Mean and SEM of three independent experiments are shown.

PML residue K394 is also conserved in the oncogenic PML-RARα fusion protein that drives APL. Intriguingly, USP22 KO NB4 human APL cells displayed an increase in the 130 kDa isoform of the PML-RARα fusion protein, compared to control NB4 cells (Fig. 3A, B). A potential transcriptional upregulation of PML-RARα mRNA could be excluded, since PML-RARα-specific PCR primers did not demonstrate significant differences in PML-RARα mRNA levels upon loss of USP22 (Fig. 3C and Supplementary Fig. 3). Comparative analysis of PML-RARα protein stability in the presence of CHX revealed PML-RARα stabilization in USP22 KO NB4 cells, compared to either WT or CRISPR/Cas9 control NB4 cells (Fig. 3D, E), which is consistent with previous findings describing basal PML-RARα stability [40].

To further analyze the dynamics of USP22-mediated PML-RARα stabilization, the long isoform of WT PML-RARα was expressed in control and USP22 KO HEK293T cells and PML-RARα stability was analyzed upon exposure to CHX. The half-life of PML-RARα was *t*_1/2_ = 5 h in control cells, but was extended to *t*_1/2_ = 10 h upon loss of USP22 (Fig. 3F, G). Interestingly, these observations agree with the increased stabilization of K394R-mutated PML-RARα compared to WT PML-RARα (Fig. 3H, I). Together, these findings identify USP22 and K394 as crucial regulator of the stability of the chimeric PML-RARα oncoprotein in APL cells.

### USP22 regulates granulocytic differentiation in APL cells

**Fig. 4:**
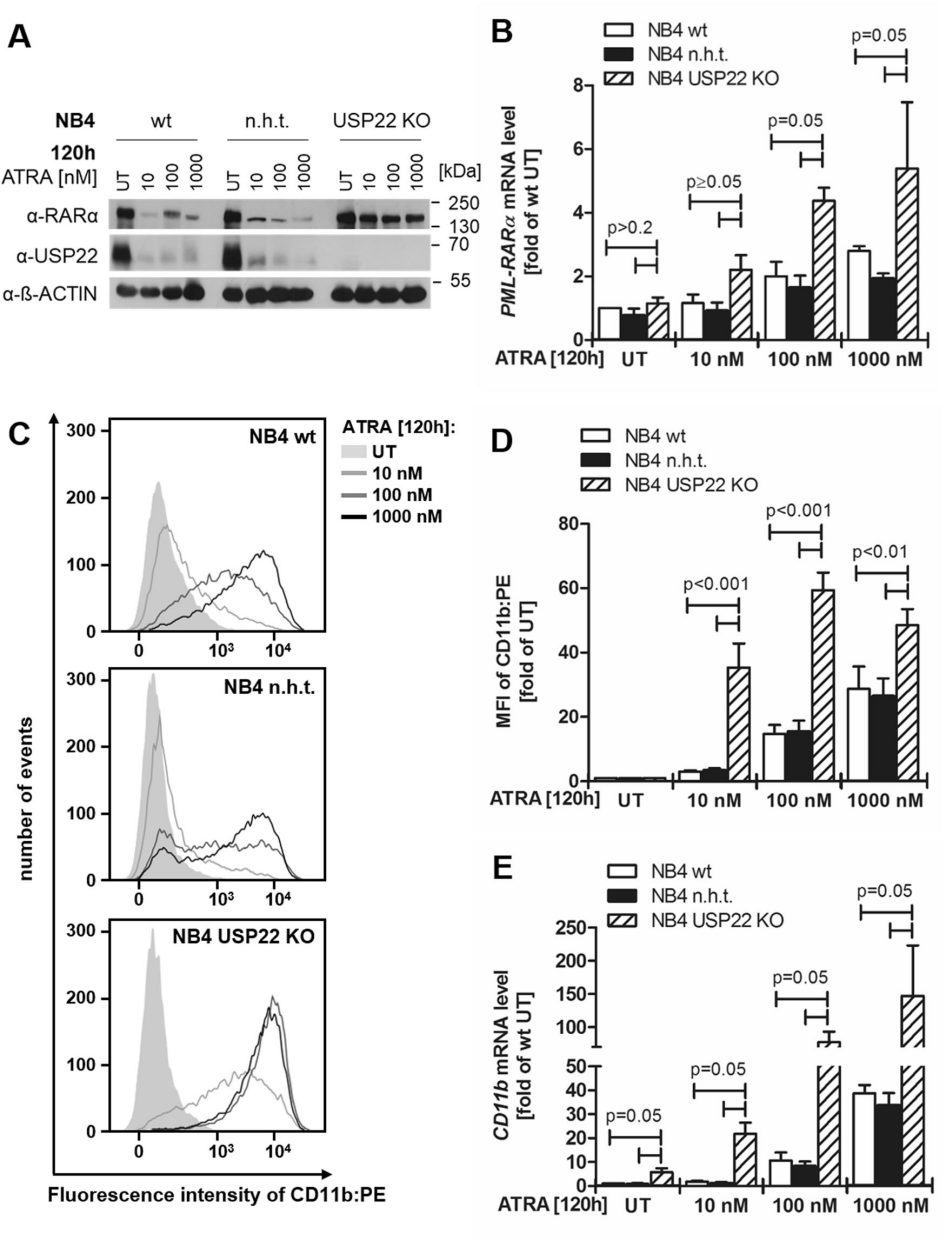
USP22 controls granulocytic differentiation of APL cells. **A** Western blot analysis of RARα and USP22 in wild-type (wt), control (non-human target; n.h.t) and USP22 knockout (KO) NB4 APL cells treated with the indicated concentrations of all-*trans* retinoic acid (ATRA) for 120 h. β-actin served as loading control. Representative blots of at least two different independent experiments are shown. **B** Basal and ATRA-induced mRNA expression levels of *PML-RARα* in wt, n.h.t and USP22 KO NB4 APL cells incubated with the indicated ATRA concentrations for 120 h using qRT-PCR. UT, untreated. Gene expression was normalized against 28S mRNA and is presented as x-fold mRNA expression compared to UT of wt NB4 cells Mean and SEM of three independent biological replicates are shown. **C** FACS analysis of wt, n.h.t., and USP22 KO NB4 APL cells incubated with the indicated ATRA concentrations for 120 h. Shown is the cell count per fluorescence intensity of PE-labeled CD11b. **D** Mean fluorescence intensities (MFIs) of CD11b-PE signals on wt, n.h.t., and USP22 KO NB4 APL cells incubated with the indicated ATRA concentrations for 120 h. UT, untreated. Mean and SEM of four independent biological replicates are shown. **E** Basal and ATRA-induced mRNA expression levels of *CD11b* in wt, n.h.t and USP22 KO NB4 APL cells incubated with the indicated ATRA concentrations for for 120 h using qRT-PCR. UT, untreated. Gene expression was normalized against 28S mRNA and is presented as x-fold mRNA expression compared to UT of wt NB4 cells Mean and SEM of three independent biological replicates are shown.

Next, we aimed to elucidate the biological relevance of USP22-mediated PML-RARα stabilization. In APL, PML-RARα acts as a transcriptional repressor of RARα target genes that are required for terminal granulocyte differentiation, e.g., transcription factors, cell cycle regulatory proteins and differentiation markers [21]. To assess the potential impact of USP22 on PML-RARα-mediated APL differentiation, WT, CRISPR/Cas9 control and USP22 KO NB4 cells were exposed to clinical and subclinical doses of ATRA, followed by immunoblotting of PML-RARα. Interestingly, loss of USP22 expression induced a prominent stabilization of PML-RARα protein levels upon ATRA incubation, compared to WT and control NB4 cells (Fig. 4A).

Of note, no significant alterations in PML-RARα mRNA expression levels could be detected in untreated WT, control or USP22 KO NB4 cells (Fig. 4B). In contrast, ATRA treatment induced PML-RARα mRNA expression and loss of USP22 potentiated the ATRA-mediated effects on PML-RARα mRNA expression (Fig. 4B). In addition, the PML-RARα protein was distinctively modified in USP22 KO NB4 cells upon ATRA-mediated receptor degradation (Supplementary Fig. 4) and an altered PML-RARα cleavage pattern with less caspase activation compared to WT and control NB4 cells was observed (Supplementary Fig. 5).

Surprisingly, by monitoring the surface expression of the CD11b differentiation marker with flow cytometry, ATRA-treated USP22 KO NB4 cells displayed a 40- to 60-fold increase in CD11b surface expression compared to untreated USP22 KO NB4 cells, while CD11b levels of WT and control NB4 cells only increased by 10- to 30-fold upon ATRA treatment (Fig. 4C, D). Strikingly, subclinical ATRA concentrations of 10 nM already resulted in a 3-fold higher CD11b surface expression in USP22 KO NB4 cells compared to WT and control NB cells, suggesting a strong USP22-mediated sensitization towards ATRA-induced differentiation. In addition, the increase in CD11b expression already occurred after 72 h ATRA treatment and gradually increased up to 120 h (Supplementary Fig. 6). Similar effects were observed by quantifying CD11b gene expression (Fig. 4E). In basal, untreated conditions, a 5-fold increase in CD11b mRNA levels could already be observed upon loss of USP22 expression, that increased to over 10- to 30-fold induction of CD11b mRNA expression upon ATRA incubation in USP22 KO NB4 cells compared to WT and control NB4 cells (Fig. 4E).

**Fig. 5:**
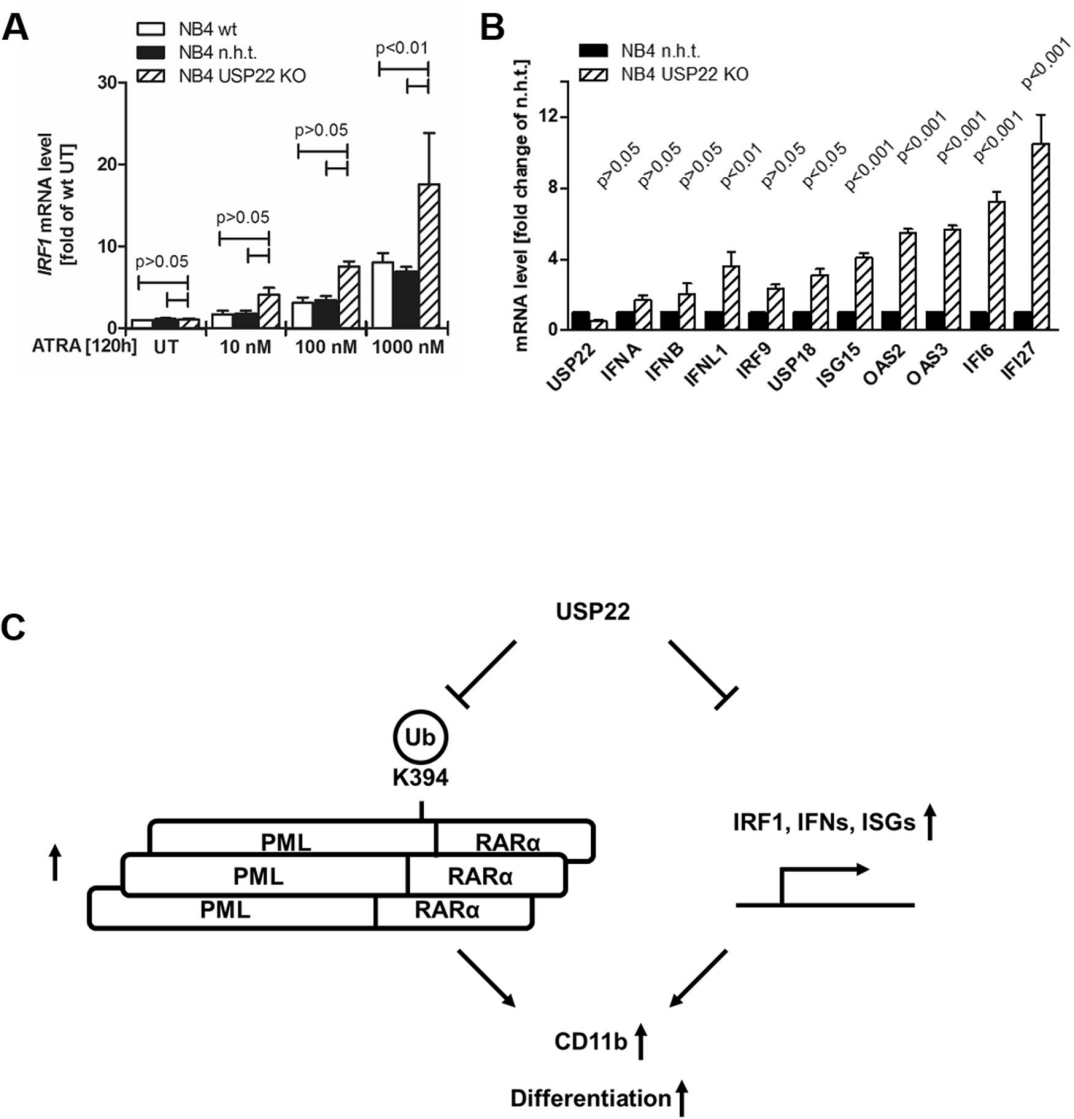
USP22 negatively regulates IFN responses in APL cells. **A** Basal and ATRA-induced mRNA expression levels of IRF1 in wild-type (wt), control (non-human target; n.h.t) and USP22 knockout (KO) NB4 APL cells incubated with the indicated ATRA concentrations for 120 h using qRT-PCR. UT, untreated. Gene expression was normalized against 28S mRNA and is presented as x-fold mRNA expression compared to UT of wt NB4 cells. Mean and SEM of three independent biological replicates are shown. **B** Basal mRNA expression levels of USP22 and the indicated ISGs in n.h.t and USP22 KO NB4 APL cells using qRT-PCR. Gene expression was normalized against 28S mRNA and is presented as x-fold mRNA expression compared to n.h.t. Mean and SEM of three independent biological replicates are shown. **C** Model of USP22-mediated effects on PML-RARα and IFN signaling in APL.

Recently, USP22 has been identified as a negative regulator of type I and III IFN signaling [41]. In agreement with previous findings [42], ATRA induced the dose-dependent upregulation of the master regulator IFN-regulatory factor 1 (IRF1) expression in NB4 cells (Fig. 5A), while loss of USP22 strongly potentiated ATRA-mediated upregulation of IRF1 (Fig. 5A). Deficiency of USP22, even in the absence of exogenous IFNs or viral infection, was sufficient to potently upregulate a wide variety of ISGs, including ISG15, 2’-5’-oligoadenylate synthetase 2 (OAS2), as well as IFN-α, -β and -λ (Fig. 5B), identifying USP22 as important negative regulator of IFN signaling and ISG expression in APL cells. Taken together, our findings reveal that USP22 controls the stability of the PML tumor suppressor and regulates APL differentiation via regulating PML-RARα stability and repressing IFNs (Fig. 5C).

## Discussion

Here, we investigate the functional interplay of the cancer-associated DUB USP22 with the master cell fate regulatory protein PML to gain deeper insights in the pro-tumorigenic functions of USP22. We demonstrate that USP22 controls basal stabilization of the tumor suppressor PML under homeostatic conditions and describe a destabilizing role of PML residue K394. Additionally, the oncogenic PML-RARα fusion protein is also stabilized in an USP22- and K394-mediated manner, through which USP22 defines sensitivity towards ATRA-mediated APL differentiation. Apart from that, we identify USP22 as negative regulator of IFN signaling in APL. Based on these findings, we suggest dual mechanistic roles of USP22 in the regulation of ATRA-mediated APL differentiation.

We describe that USP22 regulates the turnover of PML isoform IV as well as the long isoform of PML-RARα under basal conditions. In the absence of USP22, the abundance of PML and PML-RARα is increased, suggesting that USP22 enhances PML turnover. Our findings also identify PML residue K394 as determinant that controls PML protein turnover and mutating this residue stabilized PML and PML-RARα. Intriguingly, K394-ubiquitinated PML peptides have been detected in global ubiquitinome profiles upon proteasomal inhibition in several cell types [39, 43–45], suggesting a role of K394 in regulating PML levels via degradation by the 26S proteasome. Up till now, the exact mechanisms and types of ubiquitin modifications on K394 PML, and the role of USP22 here, remains to be identified. Intriguingly, our findings describe potential differences in PML mono-ubiquitination that might resemble the context-specific USP22-mediated modification of histones. As part of the Spt-Ada-Gcn5 acetyltransferase (SAGA) complex, USP22 is well-known to deubiquitinate histones H2B and H2A to regulate gene transcription, replication and DNA repair [4, 46]. In some cases, changes in histone ubiquitination patterns upon loss of USP22 are compensated by the USP22-homologous DUBs USP27X and USP51 [46], that compete with USP22 [46]. Of note, novel, non-histone USP22-dependent substrates are starting to emerge as well, including receptor-interacting serine/threonine-protein kinase 3 (RIPK3), programmed death ligand 1 (PD-L1) and estrogen receptor α (ERα) [1, 37, 47], but the role of the USP22-USP27X-USP51 triad in ubiquitinating these targets remains unclear. At present, it also remains unclear if USP22 deubiquitinates PML directly or if this occurs in cooperation with additional E3 ligases and DUBs. Similar scenarios have been described for USP13 in the regulation of STING ubiquitination through interactions with USP22 that are independent of the catalytic activity of USP22 [48, 49]. This opens the possibility that USP22 might indirectly regulate PML stability, but the underlying mechanisms remain to be identified. Intriguingly, ATO triggers the recruitment of the SUMO-dependent E3 ubiquitin ligase RNF4 to SUMOylated PML to modify SUMO chains, PML K394 and additional PML residues with ubiquitin to potentiate PML degradation [50] and ATO increases K394-ubiquitinated PML peptides [51].

An emerging concept is the pairing of DUBs with cognate E3 ligases, such as the cooperation of USP11 with the multi-subunit E3 ligase complex Roc1–Cul3–KLHL20 to control basal PML turnover [52]. Up till now, potential E3 ligases that interact with USP22 to control PML stability remain to be identified, although several E3 ligases have been described to control PML stability under basal conditions. Among these are SIAH-1 and -2 that ubiquitinate PML in the N-terminal coiled-coil region and thereby target PML as well as PML-RARα for proteasomal degradation [32]. In addition, the viral E3 ligase-like protein ICP0, expressed in herpes simplex virus type 1 (HSV-1), induces breakdown of PML NBs and PML degradation [53, 54]. Interestingly, ICP0 itself is targeted for degradation by SIAH-1 [55], demonstrating the complexity of functional consequences of post-translational modification on PML regulation and the close interplay between anti-viral as well as tumor-suppressive defense mechanisms. Up till now, the potential relevance of SIAH-1/2, or additional E3 ligases, for USP22 function and PML degradation remains to be determined.

APL therapy based on the differentiation agent ATRA typically results in remission rates over 80% [20]. Unphysiologically high therapeutic doses of ATRA alter the repressive functions of PML-RARα into genetic re-activation of genes involved in differentiation, due to ATRA-mediated replacement of co-repressors by co-activators [21]. At the same time, ATRA induces degradation of PML-RARα, which is essential for the loss of clonogenicity of leukemic blasts [25]. By re-expressing PML-RARα, ATRA-resistant human APL NB4 cells could be sensitized for ATRA-induced differentiation [56]. This strongly confirms our findings that loss of USP22 leads to PML-RARα stabilization that, upon stimulation with ATRA, potently increased CD11b expression. Intriguingly, ATRA treatment reduced USP22 levels of which the mechanisms and functional relevance remains unclear. In addition, we describe a USP22 deficiency-dependent sensitization of human APL NB4 cells for ATRA-induced differentiation caused by a prolonged PML-RARα protein stability and an upregulation of type I and III IFNs and several ISGs. In line with this, IFNα-stimulation of APL cells is known to enhance the differentiation response of APL cells towards ATRA treatment [57]. Additionally, we confirm the ATRA-induced expression of IRF1 which underlies differentiation-associated gene programs in APL [42] and further describe a substantial increase in ATRA-triggered IRF1 expression upon loss of USP22. Along with recent findings on enhanced IFN type I and III responses during viral defense in the absence of USP22 [41, 48], USP22 is revealed as major regulator of IFN signaling in human leukemia.

ATRA triggers activation of caspase-3 that cleaves PML-RARα at residue D522, followed by proteasomal degradation of the cleavage fragments [23]. We report that loss of USP22 interferes with the conversion of pro-caspase-3 into active caspase-3 with subsequent alterations of PML-RARα cleavage patterns and degradation, suggesting ATRA-dependent roles of USP22 in caspase-3 activation. Intriguingly, the conformation of PML-RARα upon ATRA binding favors the accessibility of residue D522 for caspase-3-mediated cleavage [23], while other retinoids, like etretinate, fail to induce PML-RARα degradation, presumably due to allosteric hindrance of the caspase cleavage site [25]. USP22-dependent alterations in PML-RARα K394 ubiquitination therefore might affect caspase-mediated cleavage. In hepatocellular and renal cell carcinoma however, USP22 deficiency has been reported to enhance caspase-3 activation [58, 59], emphasizing the need for further investigations.

Taken together, USP22 controls the stability of PML and PML-RARα protein as well as IFN signaling during APL differentiation. Since USP22 acts in highly context- and tumor-specific manners, our findings might open novel therapeutic opportunities to re-sensitize ATRA-resistant, relapsed APL patients to ATRA treatment by simultaneous inhibition of USP22.

## Materials and methods

### Cell lines, tissue culture and chemicals

Human colon carcinoma HT-29, human embryonic kidney HEK293T and human APL NB4 cell lines were obtained from and authenticated by DSMZ (Braunschweig, Germany). Cells were cultured in McCoys 5 A Medium GlutaMAX™-I, DMEM Medium GlutaMAX™-I or RPMI 1640 Medium, respectively, supplemented with 10% Fetal Calf Serum (FCS) and 1% penicillin/streptomycin and, in case of HEK293T, also 1% sodium pyruvate (all media and supplements from Life Technologies, Inc.). All cell lines were maintained at 37 °C in humidified incubators with 5% CO_2_ and regularly monitored for mycoplasma infections.

All chemicals and reagents used in this study were obtained from Carl Roth (Karlsruhe, Germany) or Sigma, unless stated otherwise.

### Plasmid cloning and mutagenesis

For transient expression, full-length human PML isoform IV (mRNA transcript variant 6, NM_002675.4) was subcloned into the pSBbi-blasticidin plasmid [60] (Addgene plasmid #60526) using the TOPO™ TA Cloning™ Kit (Thermo Scientific), according to manufacturer recommendations. *SfiI*-digestion sites were added to the coding sequence of PML-IV by PCR using the Platinum™ PCR SuperMix Kit (Invitrogen). For protein detection, a hemagglutinin epitope (HA-tag) was introduced at the 3’-end of PML Exon 8b after a Pro-Ser spacer. The following oligonucleotides were used: 5’-TAACTTGGCCTCTGAGGCCAGATCTAAACCGAGAATCGAAAC-3‘ and 5’-GACCTGGCCTGACAGGCCTCATCCTGCGTAATCTGGAACATCGTATGGGTAAGAAGGAATTAGAAAGGGGTG-3’. In addition, an internal *SfiI*-digestion site in PML was deleted by introducing a silent C-to-A point mutation at Gly266 using the GeneArt™ Site-Directed Mutagenesis Kit (Thermo Scientific), following the manufacturer advice. The K394R mutation in PML-IV was generated by introducing a point mutation at K394 by changing AAA to AGA, encoding for Arg394. The pSBbi-blast empty vector (EV) served as transfection control plasmid. Transient expression of the long isoform of the PML-RARα fusion protein in HEK293T cells was performed with a pSG5-PML-RARα expression plasmid, kindly provided by Hugues de Thé (Paris, France). A 6x-His-tag was inserted at the 5’-end of PML-RARα ORF using the Q5 Site-Directed Mutagenesis Kit (NEB) and following oligonucleotides: 5’-CATCACCATTCCATGGAGCCTGCACCCGCC-3’ and 5’-GTGGTGATGCATGGACCCCAGCTTAGTTTCGATTCTC-3’. The K394R mutation was introduced as described above. All plasmids generated by cloning were verified by Sanger Sequencing (Microsynth AG, Switzerland).

### CRISPR/Cas9-mediated gene inactivation

CRISPR/Cas9-mediated knockout (KO) of USP22, as well as non-human-target (n.h.t) control cell lines were generated as described previously [37]. Briefly, viral particles were generated in HEK293T cells by co-transfecting the packaging plasmids pMD2.G and psPAX2 (kind gift of Didier Trono; Addgene plasmids #12259 and #12260, respectively) with a pool of three pLentiCRISPRv2 (kind gift of Feng Zhang [61]; Addgene plasmid #52961), expressing the following sgRNA sequences to target USP22: #1: GCCATTGATCTGATGTACGG, #2: CCTCGAACTGCACCATAGGT and #3:ACCTGGTGTGGACCCACGCG. As control, a pool of three Green Fluorescent Protein (GFP)-targeting sgRNA sequences were used (Addgene plasmids #51763, #51762 and #51760) to generate control non-human target (n.h.t.) CRISPR/Cas9 cell lines. Target cells were transduced with viral particles in the presence of 8 µg/mL polybrene for 48 h, followed by puromycin selection for up to two weeks. Monoclonal cell lines were generated using limiting dilution of puromycin-resistant pools and loss of USP22 expression was verified by Western blot analysis.

### Denaturing cell lysis and immunoblotting

Whole cell lysates were prepared under denaturing conditions with the following buffer: 50 mM Tris-HCl, 1% NP-40, 0.5% sodium deoxycholate, 150 mM NaCl, 2 mM MgCl_2_, 2% sodium dodecyl sulfate (SDS), 2 mM dithiothreitol (DTT), EDTA-free Protease Inhibiter Cocktail (PIC; Roche) and 250 U/mL Pierce Universal Nuclease (Thermo Scientific). For complete solubilization of insoluble proteins, lysates were subjected to boiling for 5 min at 96 °C in 6x SDS loading buffer (350 mM Tris Base pH 6.8, 38% glycerol, 10% SDS, 93 mg/ml dithiothreitol (DTT), 120 mg/ml bromophenol blue) prior to SDS-PAGE and Western blot analysis. The following primary antibodies were used in this study: rabbit-anti-PML (ab179466, Abcam), rabbit-anti-USP22 (ab195298, Abcam), mouse-anti-β-ACTIN (A5441, Sigma-Aldrich), mouse-anti-GAPDH (5G4-6C5, HyTest, Ltd., Turku, Finland), mouse-anti-HA (F-7) (sc-7392x, Santa Cruz Biotechnology), mouse-anti-Vinculin (V9131, SIGMA-Aldrich), rabbit-anti-GFP (632592, Clontech), rabbit-anti-RFP (ab62341, Abcam), rabbit-anti-RARα (62294 S, Cell Signaling), rabbit-anti-His-tag (sc-53073, Santa Cruz Biotechnology), mouse-anti-ubiquitin (P4D1) (sc-8017, Santa Cruz Biotechnology). Protein detection was performed by incubation with horseradish peroxidase (HRP)-conjugated goat anti-mouse IgG and goat anti-rabbit IgG (Santa Cruz Biotechnology) secondary antibodies, followed by detection with enhanced chemiluminescent reagent (Amersham Bioscience, Freiburg, Germany).

### qRT-PCR

Gene expression analysis was performed using quantitative real-time (qRT) PCR on total RNA isolated from the indicated cell lines with use of the peqGOLD Total RNA Kit (Peqlab) as proposed by the manufacturer. Reverse transcription of 1 µg of isolated RNA was done according to the manufacturer recommendations (RevertAid H Minus First Strand cDNA synthesis Kit; Thermo Scientific). qRT-PCR analysis was performed in 40 cycles with the use of the SYBR® Green PCR Master Mix (Applied Biosystems) with the QuantSudio™ 7 Flex Real-Time PCR System (Applied Biosystems) in MicroAmp™ optical 384-Well reaction plates (Applied Biosystems) at a final concentration of 250 nM. The following oligonucleotides were used: pan-human PML: 5’-GACTTCTGGTGCTTTGAGTGCGAG-3’ and 5’-GCTCACTGTGGCTGCTGTCAAG-3’; PML-RARα (long form): 5’-GCCCCGTCATAGGAAGTGAG-3’ and 5’-TGACCCCATAGTGGTAGCCT-3’; hCD11b: 5’-ACTTGCAGTGAGAACACGTATG-3’ and 5’-AGAGCCATCAATCAAGAAGGC-3’; IRF1: 5‘-ACAGCACCAGTGATCTGTACAAC-3’ and 5’-TTCCCTTCCTCATCCTCATCT-3’ and 28s rRNA as internal reference: 5’-TTGAAAATCCGGGGGAGAG-3’ and 5’-ATTGTTCCAACATGCCAG-3’. All other primers are described in [41]. Quantification of gene expression levels was performed using the 2^-ΔΔCT^ method [62] after normalization to 28s rRNA expression. Experiments were performed as technical triplicates and biological replicates and presented as the 2^-ΔΔCT^-transformed fold value.

### Transient transfections

For transient transfection, HEK293T cells were seeded in antibiotic-free culture medium at a density of 0.3 × 10^5^ per cm² at 24 h prior to transfection with 2 µg of PML-encoding plasmid DNA per 1 × 10^6^ cells with FuGENE HD transfection reagent (Promega) at a ratio of 3:1. As transfection controls, the indicated cells were co-transfected with GFP or mCherry plasmids (Clontech) at a concentration of 0.6 µg DNA per 1 × 10^6^ cells.

### Cycloheximide chase experiments

The indicated cell lines were seeded 24 h prior to incubation with 20 µg/mL cycloheximide (CHX, SIGMA-Aldrich) for different timepoints, followed by PBS washing and denaturing cell lysis as described. Comparison of protein levels on Western blots was assessed by quantification of the gray-level intensities of proteins of interest and normalization to reference proteins of the same blot using ImageJ software (v1.52e).

### Immunoprecipitations and affinity pulldown experiments

HEK293T cells were seeded at a density of 0.5 × 10^5^ cells per cm² and transfected with 1 µg plasmid DNA per 1 × 10^6^ cells as described above. 24 h post transfection, cells were washed three times with ice-cold PBS, supplemented with 25 mM N-ethylmaleimide (NEM; SIGMA-Aldrich). Washed cell pellets were resuspended in lysis buffer (20 mM Tris, 1% NP-40, 50 mM NaCl, 10% Glycerol, 5 mM EDTA, pH 7.5) supplemented with 1% SDS, 25 mM NEM, PIC, 1 mM Sodium orthovanadate, 5 mM Sodium fluoride, 1 mM ß-glycerophosphate and 250 µ/mL Pierce Universal Nuclease (Thermo Scientific). Lysates were subjected to 2 × 10 s. pulse sonification at 40% amplitude for complete shearing of genomic DNA and breakup of insoluble protein aggregates. Prior to overnight bead incubation, 3 mg of protein lysates were diluted at least 1:10 in lysis buffer without supplements to decrease the SDS concentration to <0.1%. After that, beads were washed six times with lysis buffer without supplements. For immunoprecipitation of HA-tagged PML, anti-HA magnetic beads (Pierce, Thermo Fisher) were incubated and washed as described above. Finally, proteins of interest were eluted from the beads by boiling at 96 °C for 5 min in 2 x Laemmli sample buffer, followed by Western blot analysis.

### Flow cytometric analysis

NB4 cells were seeded at a density of 0.1 × 10^6^/mL one day prior to treatment. All-*trans* retinoic acid (ATRA; SIGMA-Aldrich) was dissolved in DMSO (stock concentration: 10 mM) and diluted in culture medium to 0.1 mM and added to the cells at the indicated final concentrations for 120 h. Cells were then collected, washed and blocked in PBS/3% FCS for 30 minutes on ice. For direct fluorophore labeling, samples were incubated with PE-conjugated CD11b-mouse-IgG1 antibody (BD Pharmingen) in a 1:40 dilution for 30 min on ice, washed again and directly subjected to flow cytometric analysis on a BD FACS Canto II device (BD Biosciences) with DIVA software (v6.1.3). Per sample, 10,000 events were acquired after scatter-dependent doublet and debris exclusion from technical duplicates. Statistical analysis of biological triplicates was performed on the normalized Mean Fluorescence Intensity (MFI) changes of the PE-fluorescence signal of treated samples, compared to the MFI of untreated samples. Population graphs were processed with FlowJo software (v10.6.2).

### Caspase activity assay

Relative caspase-3 and -7 activity was assessed using the CellEvent™ Caspase-3/7 Green Detection Reagent as suggested by the manufacturer. In detail, the detection reagent is a fluorophore-conjugated peptide harboring the caspase-3- and -7-specific cleavage motif DEVD (Asp-Glu-Val-Asp). Upon caspase activation, the peptide is cleaved and the fluorescent signal becomes released as detectable signal in the FITC channel. Combined with the indicated treatments, 2 µM reagent was co-incubated throughout the treatment duration in 96-well plates. Caspase activity was monitored and quantified using an ImageXpress® Micro XLS Widefield High-Content Analysis System and MetaXpress® Software (Molecular Devices Sunnyvale, CA, USA). Total nuclei numbers were determined as reference using 1 µg/mL Hoechst-33342.

### Statistical analysis

All experiments were performed as at least 3 biological replicates and data are shown as means ± SEM. Statistical analysis was done with GraphPad Prism (v7) applying regular unpaired 2-way ANOVA and Sidak’s multicomparison post hoc test. *P* values indicated in CHX-quantification graphs describe the significance between variances of the compared cell lines.

### Supplementary information

Supplementary Materials

Original Data File

## Data Availability

The data supporting the findings of this study are available from the corresponding author upon reasonable request.
