# Supplementary material for: USP22 regulates APL differentiation via PML-RARα stabilization and IFN repression
Source: Cell Death Discov. 2024 Mar 11;10:128. doi: 10.1038/s41420-024-01894-8 (PMC10928094; doi:10.1038/s41420-024-01894-8)
Supplement: Supplementary file 1 — Supplementary Materials [file 41420_2024_1894_MOESM1_ESM.pdf]

Supplemental figure legends

USP22 regulates APL differentiation via PML-RARα stabilization and IFN repression

Lisa Kowald, Jens Roedig, Rebekka Karlowitz, Kristina Wagner, Sonja Smith, Thomas Juretschke, Petra Beli, Stefan Müller and Sjoerd J. L. van Wijk

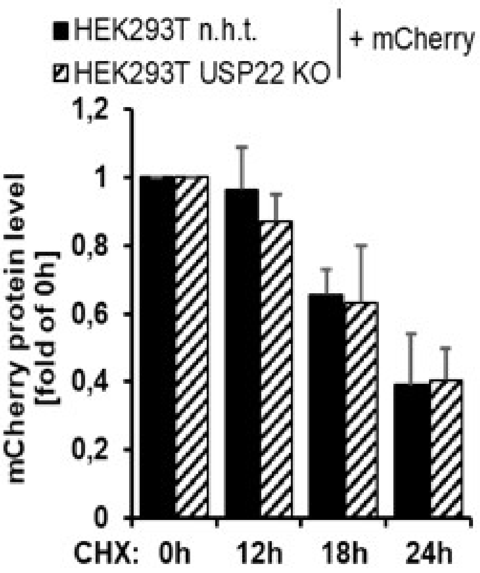

Supplemental Figure 1: Densitometric quantification of grey level intensities of mCherry expression detected by Western blot analysis in control (non-human target; n.h.t) and USP22 knockout (KO) HEK293T cells in the presence of cycloheximide (CHX) for the indicated timepoints, normalized against loading control intensities. Mean and SEM of three independent biological replicates are shown.

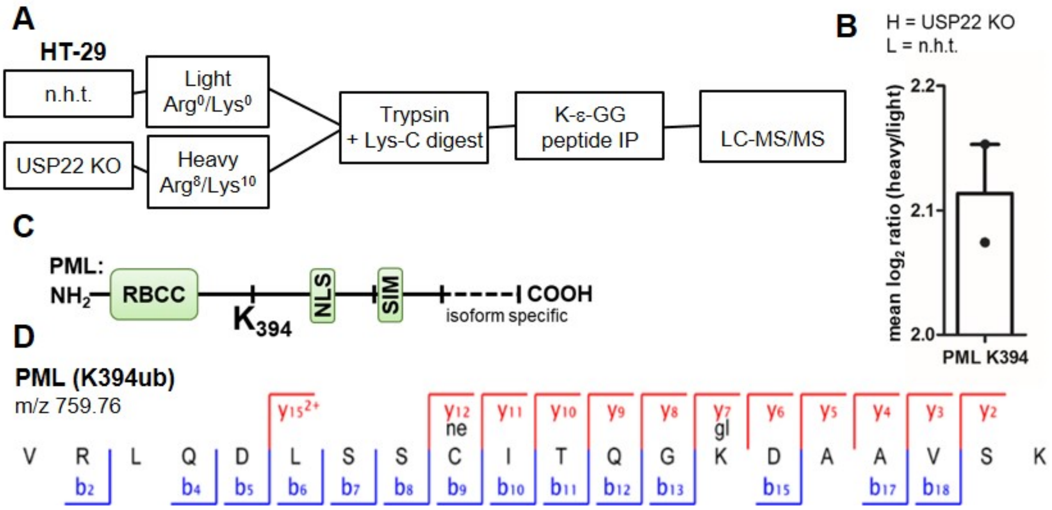

Supplemental Figure 2: USP22-dependent ubiquitination of PML K394. **A.** Schematic overview of the SILAC procedure to identify USP22-dependent alterations in the cellular ubiquitinome (published in (35)). **B.** MaxQuant-based calculations of the promyelocytic leukemia (PML) K394 peptide abundance, based on two biological replicates of SILAC-labeled HT-29. The Andromeda score of the PML K394 site was 183,09 with a PEP = 4,45867E-53. **C.** Schematic representation of the PML domain structure. RBCC: RING B-box coiled-coil; NLS: Nuclear Localization Signal; SIM: SUMO-interacting motif. **D.** Representative m/z mass-charge ratio of the ion spectrum of the PML peptide sequence comprising a di-Gly-remnant at position K394 identified in USP22 KO HT-29 cells, compared to CRISPR/Cas9 control HT-29 cells.

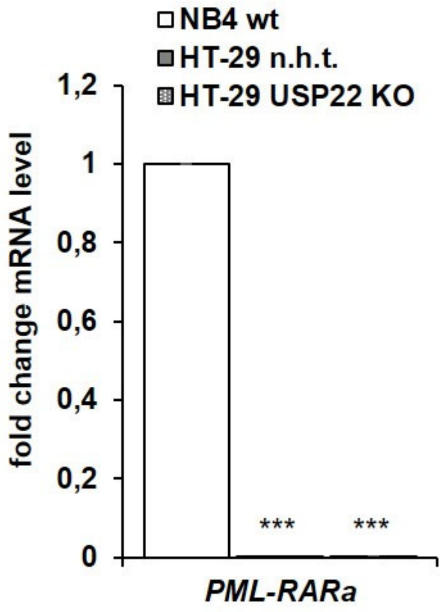

Supplemental Figure 3: Validation of qRT-PCR oligonucleotides to detect the long isoform of PML-RARα in NB4 and HT-29 control (non-human target; n.h.t) and USP22 knockout (KO) cell lines. Mean and SEM of three independent biological replicates are shown.

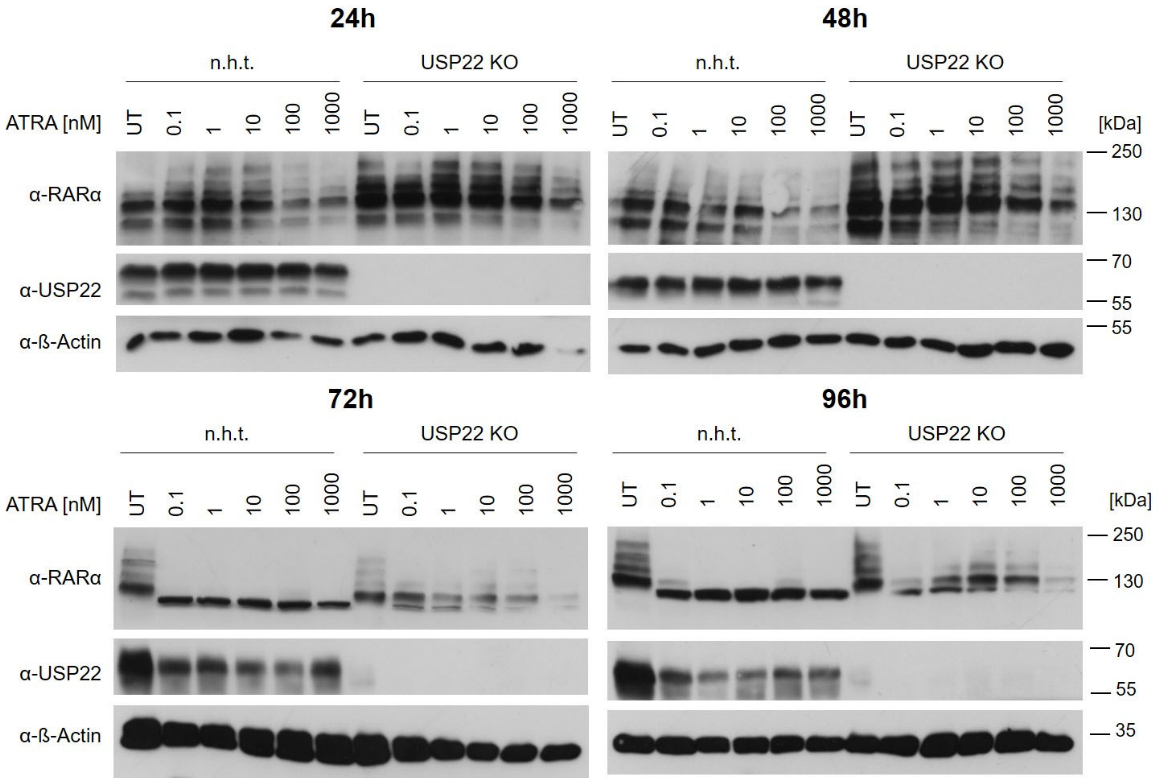

Supplemental Figure 4: Western blot analysis of RARα and USP22 expression in control (non-human target; n.h.t) and USP22 knockout (KO) NB4 acute promyelocytic leukemia (APL) cells treated with the indicated amounts of all-*trans* retinoic acid (ATRA) for the indicated timepoints. β-Actin served as loading control. Representative blots of at least two different independent experiments are shown.

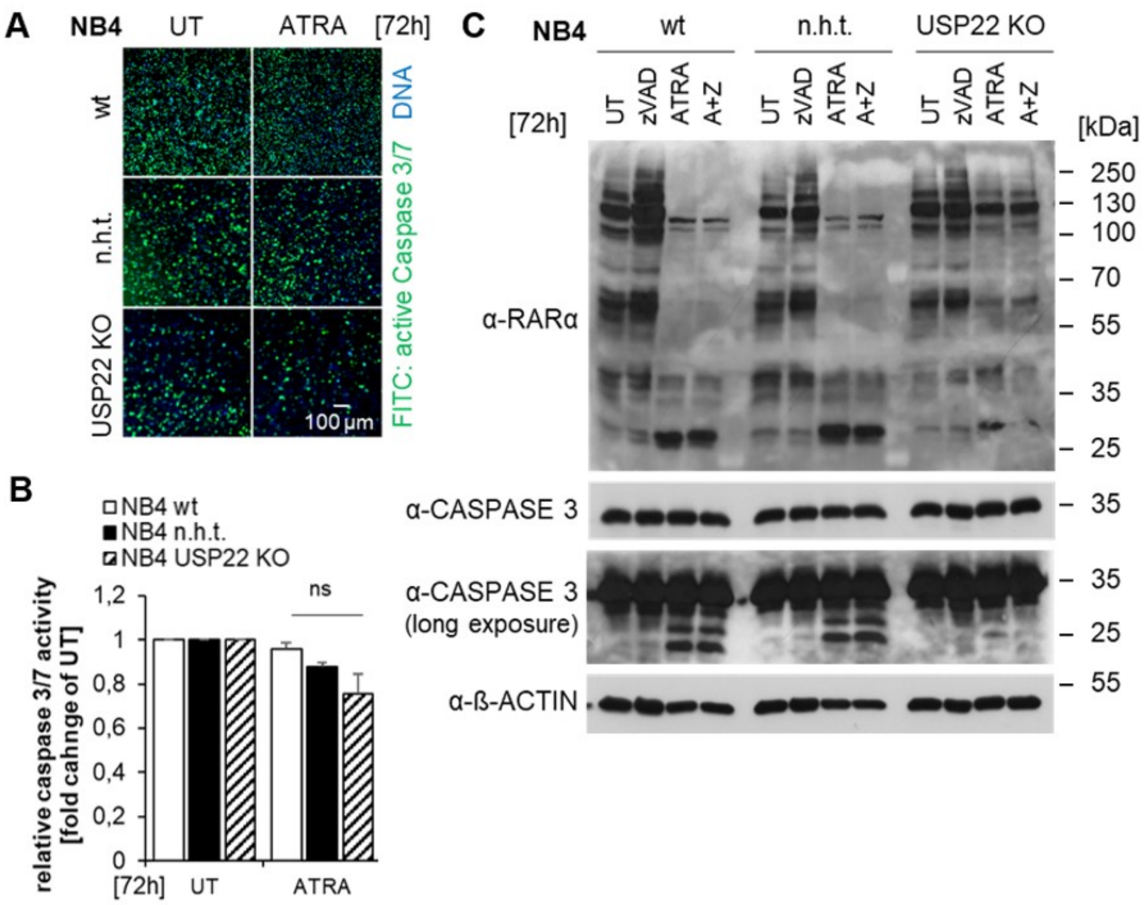

Supplemental Figure 5: A. Representative imaging-based quantification of caspase-3/7 activity in NB4 wild-type (wt), control (non-human target; n.h.t) and USP22 knockout (KO) cells, incubated with 100 nM ATRA for 72 h with 1 µM Caspase-3/7-detection reagent (FITC; green) and nuclear counterstaining (Hoechst; blue). **B.** Determination of caspase-3/7 activity in wt, n.h.t. and USP22 KO NB4 APL cells incubated with 100 nM ATRA for 72 h as fraction of FITC-positive nuclei relative to total nuclei and normalized to untreated (UT). **C.** Western blot analysis of wt, n.h.t and USP22 KO NB4 APL cells treated with the indicated amounts of zVAD.fmk (20 µM) and ATRA for the indicated timepoints. β-Actin served as loading control. Representative blots of at least two different independent experiments are shown.

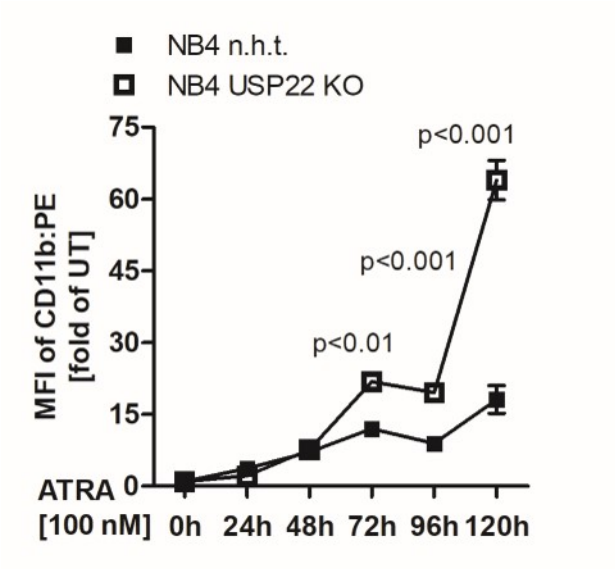

Supplemental Figure 6: Mean fluorescence intensities (MFIs) of CD11b-PE signals of control (non-human target; n.h.t) and USP22 knockout (KO) NB4 APL cells incubated with the 100 nM ATRA for the indicated periods. Data are presented as fold over untreated (UT) and mean and SEM of three independent biological replicates are shown.
