# Supplementary material for: USP22 regulates APL differentiation via PML-RARα stabilization and IFN repression
Source: Cell Death Discov. 2024 Mar 11;10:128. doi: 10.1038/s41420-024-01894-8 (PMC10928094; doi:10.1038/s41420-024-01894-8)

Figure 1A

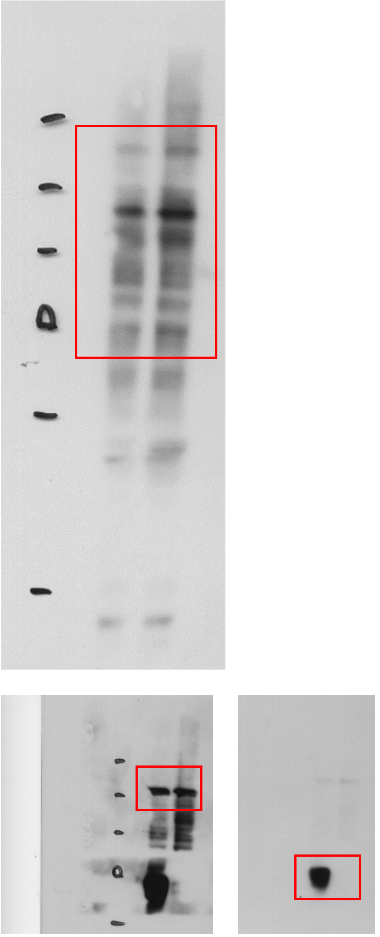

high exposure

low exposure

Figure 1D

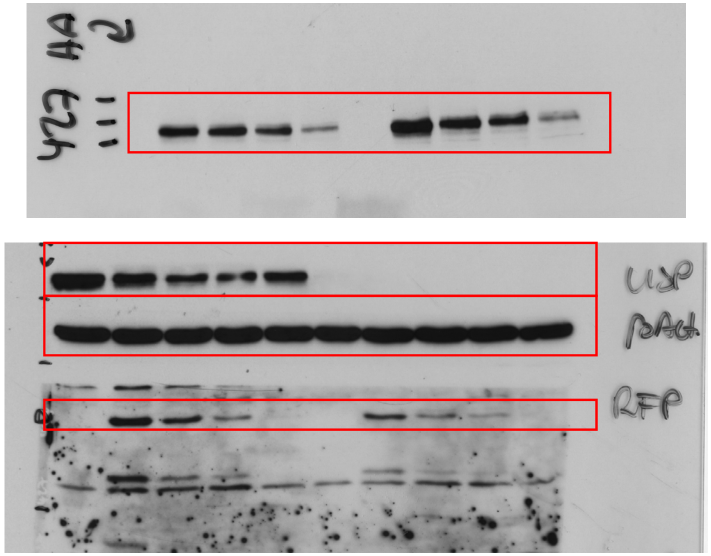

Figure 2B

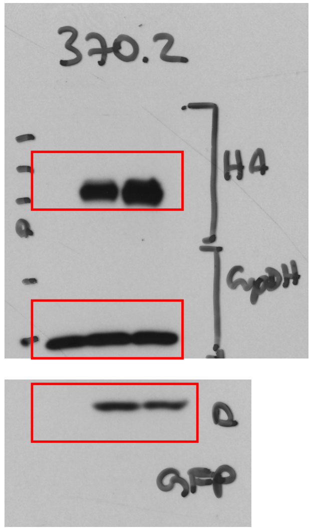

Figure 2C

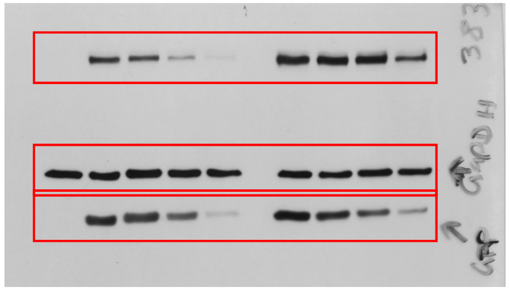

Figure 2E

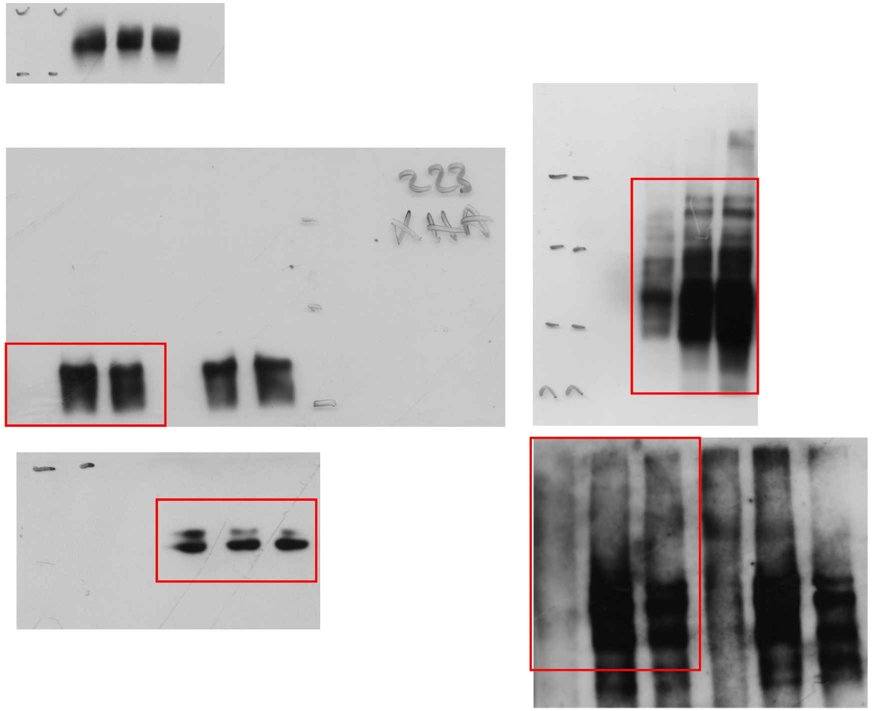

Figure 3A

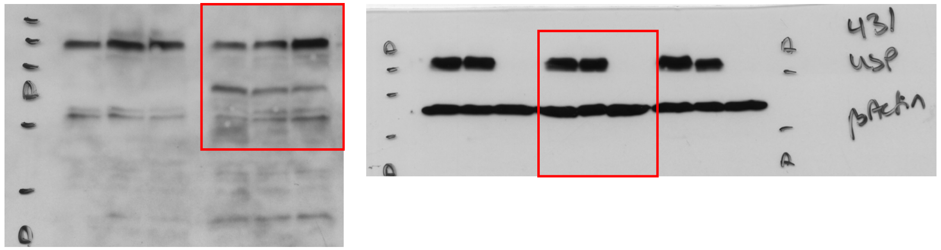

Figure 3D

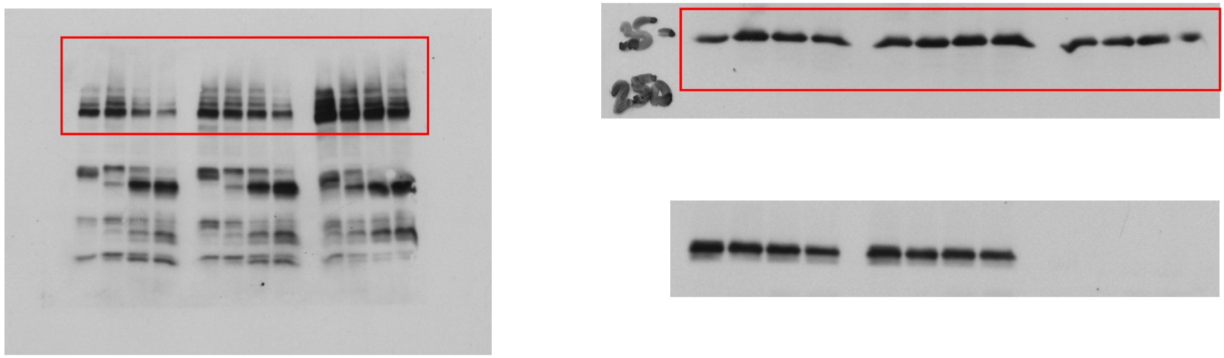

Figure 3F

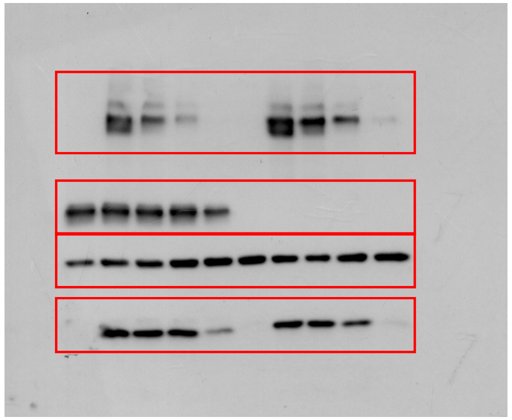

Figure 3H

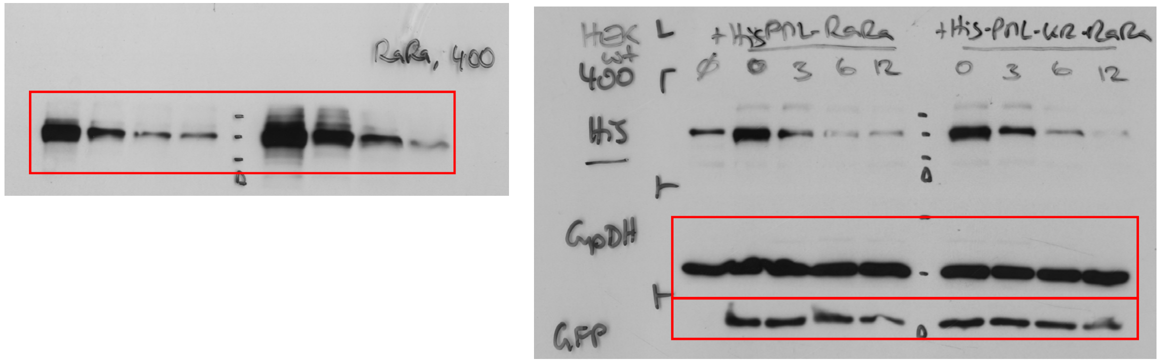

Figure 4A

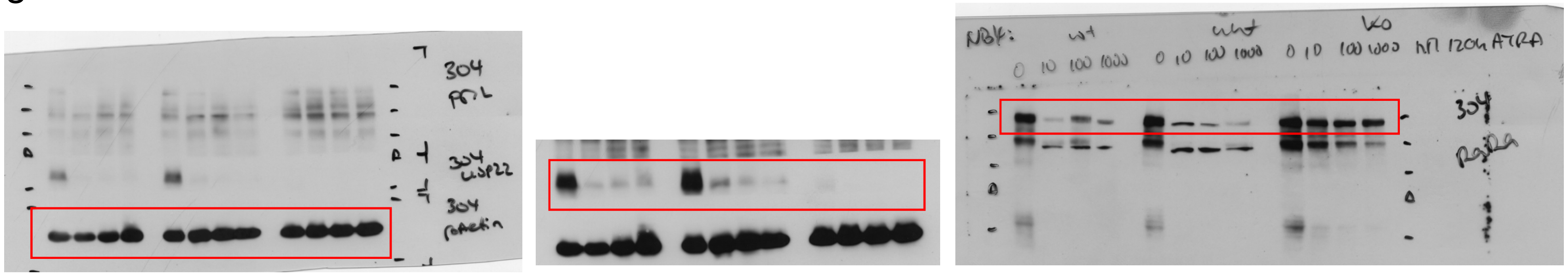

Figure S4A

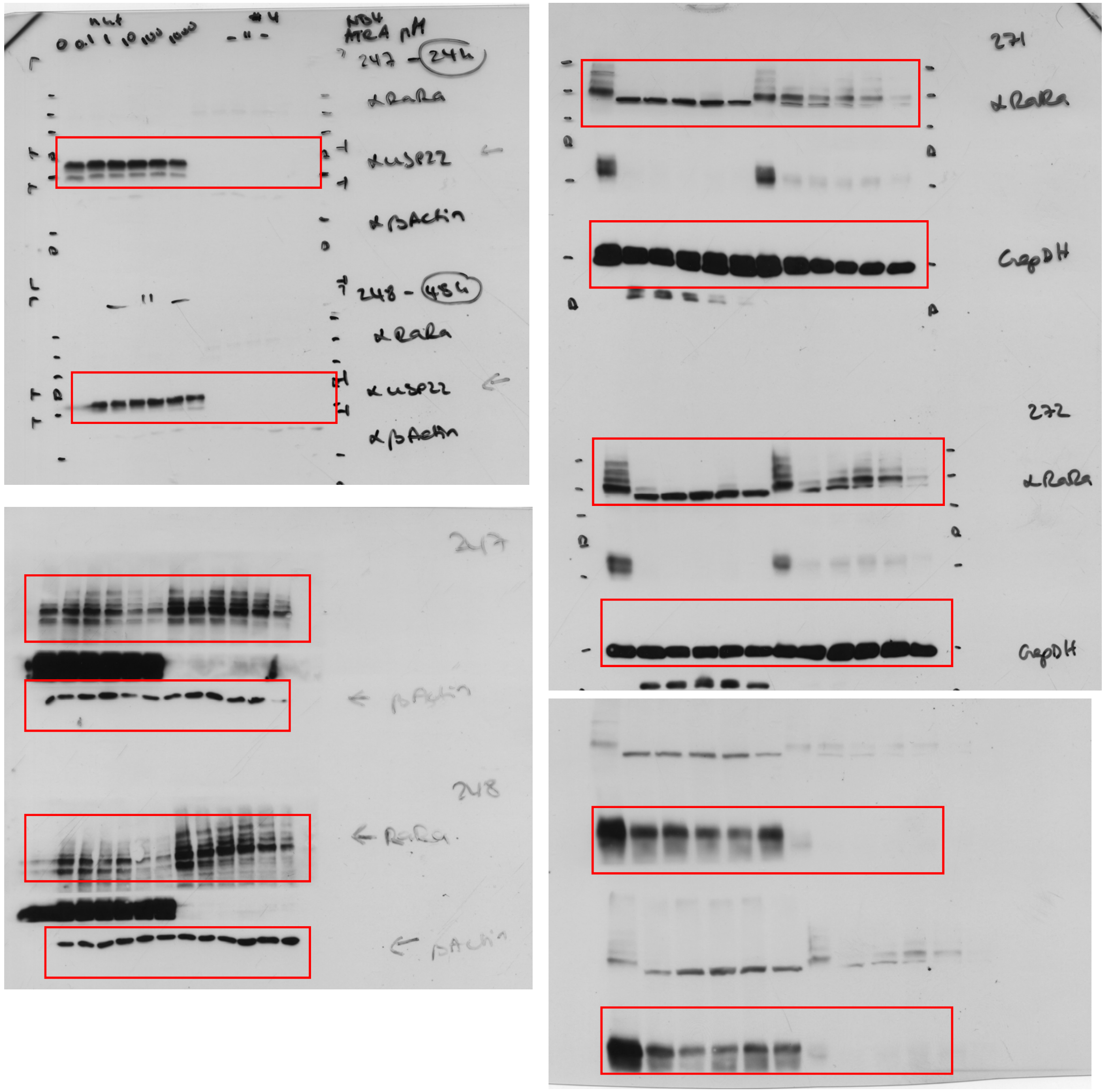

Figure S5C

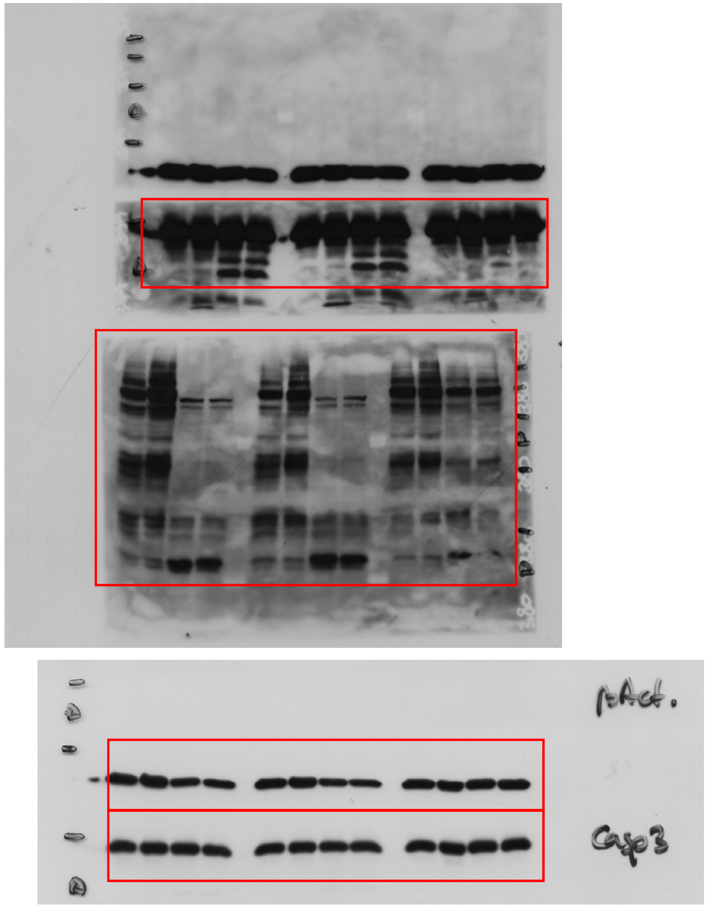

Supplement: Supplementary file 2 — Original Data File [file 41420_2024_1894_MOESM2_ESM.pdf]
